# Clinical guidelines and planning for orthodontic-surgical treatment using clear aligners

**DOI:** 10.1590/2177-6709.30.1.e25spe1

**Published:** 2025-04-07

**Authors:** Arthur CUNHA, Henrique Martins da SILVEIRA, José Augusto Mendes MIGUEL

**Affiliations:** 1 State University of Rio de Janeiro (UERJ), Orthodontic clinic (Rio de Janeiro/RJ, Brazil).; 2 State University of Rio de Janeiro (UERJ), Division of Oral and Maxillofacial Surgery, Pedro Ernesto University Hospital (Rio de Janeiro/RJ, Brazil).

**Keywords:** Orthognathic surgery, Removable orthodontic appliances, Patient care planning, Treatment outcome, Cirurgia ortognática, Alinhadores estéticos, Plano de tratamento, Resultados do tratamento

## Abstract

**Introduction:**

Orthognathic surgery, a well-established procedure for correcting significant skeletal imbalances, addresses both aesthetic and functional concerns. Traditionally, fixed orthodontic appliances have been preferred due to their effectiveness in managing dental movements and ensuring postoperative stability. However, the increasing demand for more aesthetic and comfortable options has led to the growing use of clear aligners, even in complex surgical cases. Although aligners present some limitations, such as reduced control over certain dental movements and the need for greater patient compliance, they offer notable advantages in terms of comfort and aesthetics. Similar to fixed appliances, the successful integration of aligners with orthognathic surgery requires meticulous planning and close coordination between the orthodontist and surgeon.

**Objective:**

This article provides detailed guidelines for professionals on planning and executing orthodontic-surgical treatments using clear aligners, covering both conventional and surgery-first approaches. Clinical examples will illustrate the aesthetic and functional benefits, as well as key technical challenges in daily practice.

## RESUMO

**Introdução:**

A cirurgia ortognática é uma prática bem estabelecida, destinada ao tratamento de casos de desequilíbrios esqueléticos significativos que comprometem a estética e a função. Tradicionalmente, os aparelhos ortodônticos fixos são a escolha predominante para tratamentos ortodôntico-cirúrgicos, devido à sua eficácia no controle dos movimentos dentários e à estabilidade que proporcionam no pós-operatório. No entanto, a crescente demanda por opções de tratamento mais estéticas e confortáveis tem incentivado o uso de alinhadores ortodônticos, mesmo em casos complexos como os cirúrgicos. Embora os alinhadores apresentem algumas limitações, como menor controle sobre certos movimentos dentários e a necessidade de maior adesão do paciente, seu uso em terapias ortodôntico-cirúrgicas oferece algumas vantagens, como maior conforto e estética durante o tratamento. Assim como quando utilizamos aparelhos fixos, a combinação de alinhadores com cirurgia ortognática exige um planejamento cuidadoso e uma forte integração entre ortodontista e cirurgião, para alcançar resultados previsíveis.

**Objetivo:**

Este artigo visa fornecer propostas detalhadas de planejamento e execução de tratamentos ortodôntico-cirúrgicos com alinhadores transparentes, abordando tanto o preparo convencional quanto o protocolo do benefício antecipado. Serão apresentados exemplos clínicos de ambas as abordagens, destacando os benefícios estéticos e funcionais, bem como as considerações técnicas necessárias para a sua implementação eficaz na prática clínica diária.

## INTRODUCTION

Since the late 20^th^ century, orthognathic surgery has achieved remarkable success in correcting facial deformities, driven by advances in techniques and technologies. These improvements have allowed for precise corrections of a wide range of skeletal and dentofacial issues, significantly enhancing both functional outcomes and facial aesthetics, particularly in severe cases. As a result, patients have experienced notable improvements in their quality of life.^1^ Over time, technological innovations have further refined these procedures, transitioning from traditional 2D cephalometric radiographs to more sophisticated 3D analyses and surgical planning with cone-beam computed tomography (CBCT). Additionally, CAD/CAM technology has revolutionized the production of surgical splints, increasing the precision and predictability of outcomes. One of the most transforming advancements in recent years is the Virtual Surgical Planning (VSP), which has dramatically reduced preoperative planning time, shortened surgery duration, and improved the accuracy of osteotomies and fixation techniques.^2^

Traditionally, fixed orthodontic appliances have been the primary choice for orthognathic treatment, offering intermaxillary fixation and stabilizing the dental arches during both trans- and post-operative periods. However, concerns over the aesthetics of brackets and the difficulty in maintaining oral hygiene often prevent adult patients from choosing this treatment option.^3^ ^,^ ^4^ Consequently, the demand for clear aligners has grown. In addition to being more discreet, aligners have been shown to facilitate better oral hygiene, potentially reducing the risk of cavities and periodontal diseases associated with prolonged use of fixed appliances.^5^ Initially, clear aligners were used to correct minor malocclusions, such as mild misalignments or small diastemas.^6^ With advancements in materials and digital treatment planning, their use has expanded to more complex cases, including those requiring surgical intervention, where results are comparable to those achieved with fixed appliances.^7^

Despite these advancements, the scientific evidence supporting the efficacy of aligners remains limited, even after over two decades of use. Most of the available data comes from clinical reports, case series, and expert opinions,^8^ which are insufficient to definitively confirm many of the benefits highlighted by manufacturers and leading opinion formers. While there has been an increase in studies with more rigorous methodologies, many publications still exhibit limitations. Systematic reviews advise caution in interpreting the results due to the small number and variability of studies. To solidify the evidence base, more comprehensive, long-term studies are needed to validate the advantages of aligners in surgical contexts.^9^

Some limitations have been reported on the use of aligners, including difficulties with root and extrusive movements, inadequate correction of the sagittal intermaxillary relationship, and dependence on patient compliance. Additionally, aligners demonstrate reduced efficacy in closing extraction spaces, achieving stable occlusal contacts, and facilitating sufficient arch expansion, compared to fixed appliances.^10^ The current literature suggests that aligners are more effective for mild to moderate cases without significant rotations or vertical movements, unless combined with auxiliary devices such as attachments, buttons, elastics, and skeletal anchorage. For more complex cases, fixed appliances still appear to offer better outcomes.^11^ Consequently, many orthodontists may hesitate to adopt aligners in orthognathic surgery due to their unfamiliarity with the technique, while surgeons may be reluctant to proceed without the use of fixed appliances during surgery. Moreover, the combination of aligners with orthognathic surgery remains under-researched, with most available studies being case reports or expert opinions focused on fixation methods and clinical techniques. More robust research, such as randomized controlled trials and long-term studies, is needed to establish definitive protocols and confirm the efficacy of aligners in surgical contexts.^12^

The first publication on orthognathic treatment with aligners appeared in 2005.^13^ At that time, limitations such as reduced control over dental rotations led the author to use segmented mechanics with fixed appliances to address these challenges in one patient. Additionally, in both cases, aesthetic fixed appliances with passive archwires were installed three months before surgery, following dental decompensation achieved by the aligners. A retrospective study later evaluated the accuracy of dental movements during the pre-surgical orthodontic phase using 3D superimpositions, comparing simulated movements with clinical results. The authors concluded that aligners are a viable option for pre-surgical preparation due to their precision in leveling the arch and achieving dental decompensation.^9^ Further research analyzed the occlusal outcomes of patients with dentofacial deformities treated with orthognathic surgery and aligners, showing a significant improvement in post-treatment PAR (Peer Assessment Rating) index scores, which measures the alignment and occlusion of the teeth.^14^ In a randomized clinical trial, patients treated with clear aligners demonstrated better outcomes in periodontal health and quality of life compared to those treated with traditional fixed appliances, reinforcing the potential benefits of aligners in surgical cases.^15^

A retrospective study investigating potential differences between the use of clear aligners and fixed appliances during surgery found no significant differences in surgery duration, the need for dental extractions or grafts, post-operative swelling, or periodontal health.^7^ In a 2024 randomized clinical trial evaluating the efficacy and skeletal stability of combining the surgery-first approach with clear aligners versus the conventional orthognathic protocol, both groups demonstrated acceptable skeletal stability six months post-surgery.^16^ The accuracy of VSP in surgical treatments using aligners was found to be similar to that achieved with fixed appliances, as no significant differences were identified between the two methods. These findings suggest that clear aligners can offer a viable alternative to fixed appliances in orthognathic surgery without compromising clinical outcomes.^17^

While using aligners in orthognathic surgery poses certain challenges, orthodontists are increasingly inclined to adopt them due to growing patient demand for more aesthetic, hygienic, and comfortable treatments without metal components. Recent technological advancements, such as improved 3D treatment planning and more precise aligner designs, have further enhanced this approach. To enable meaningful comparisons between outcomes using fixed appliances and this newer therapeutic option, it is essential to standardize clinical procedures. The present article aims to provide guidelines for planning and executing orthognathic surgery with clear aligners, offering a practical and reproducible protocol that can be used in future studies and clinical practice.

## VIRTUAL SURGICAL PLANNING (VSP)

**Figure 1:**
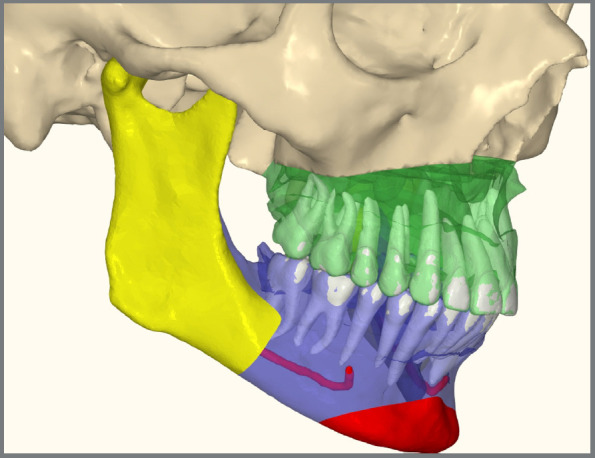
Virtual Surgical Planning using NemoStudio software (Leganés, Madrid, Spain) allows for an initial case evaluation. At this stage, the clinician assesses the optimal jaw positioning, ensuring a balanced integration of the patient’s aesthetic and functional goals.

Virtual Surgical Planning (VSP) begins with comprehensive orthodontic exams, including photographic records, panoramic and lateral radiographs, intraoral scans, and cone-beam computed tomography (CBCT). The primary goal is to conduct an early and thorough analysis to align the objectives of both the orthodontist and the surgeon. VSP is divided into three key phases: cephalometric analysis, which evaluates skeletal structures and relationships; model analysis, which examines dental alignment and occlusion; and facial analysis, which assesses overall facial aesthetics. This integrated approach ensures that both functional occlusion and facial harmony are addressed without compromise. The VSP is guided by cephalometric standards, focusing on facial balance, airway integrity, and surgical feasibility (Fig. 1). However, one of the major challenges in VSP is accurately positioning the maxillomandibular complex while maintaining aesthetic harmony along multiple spatial axes. Achieving a “normal” Class I occlusion with correct overbite and overjet may be straightforward, but determining the precise positioning of the maxillomandibular structures across vertical, horizontal, and rotational axes (pitch, yaw, and roll) without affecting facial balance remains complex. Although digital advancements have improved surgical planning, certain aspects, such as refining the integration of facial aesthetics with functional outcomes, still require further development.

**Figure 2:**
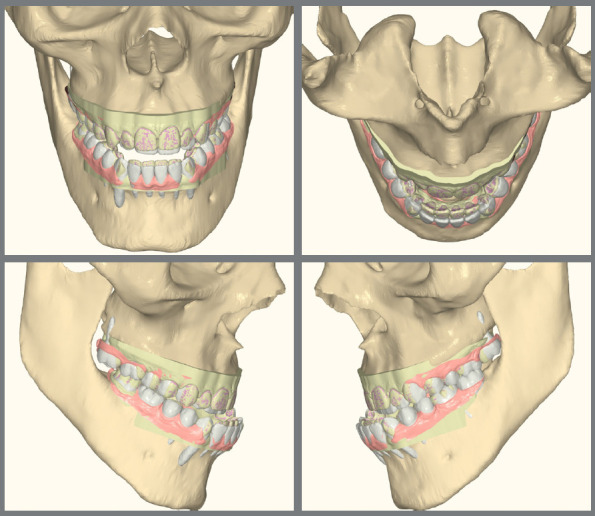
Digital setup includes the study of arch forms, Bolton analysis, curve of Spee, and anteroposterior and transverse movements. This process helps determine the need for extractions or interproximal reduction. The initial tooth positions (in beige) are shown in contrast to the predicted final positions (in white), simulating the required dental decompensations for the case.

A crucial step in planning is determining the final position of the lower incisors. This prediction is made by adding the dental or model discrepancy (MD), which is derived from model analysis, to the cephalometric discrepancy (CD), which calculates the space needed for ideal anterior tooth positioning. The total discrepancy (TD), obtained by summing MD and CD, defines the projection of the lower incisors. Similar to Tweed’s analysis, changes to the IMPA angle can be interpreted as the creation of space in the dental arch when the incisors are projected forward, or a reduction in space when the incisors are retracted.^18^ Model analysis further estimates the space required to correct malocclusion by evaluating factors such as arch form, Bolton analysis, the curve of Spee, and anteroposterior and transverse movements. These measurements guide orthodontic decisions regarding extractions, interproximal reductions, or tooth inclination to create adequate space (Fig 2). It is important to note that software used to predict these movements may sometimes underestimate the accuracy of certain adjustments, requiring careful verification by the orthodontist.^19^

## PRE- AND PERIOPERATIVE CARE

**Figure 3:**
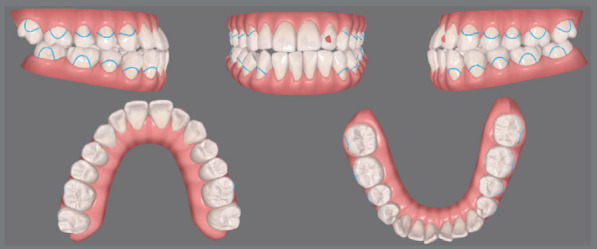
Initial setup with Clincheck^®^ software (Align Technology, Santa Clara, Calif) demonstrates that before the surgical procedure, the aligners should be passive, without attachments, and with cutouts for molars, premolars, and canines, to allow for the bonding of buttons that will be used during intermaxillary fixation and post-surgical stabilization.

**Figure 4:**
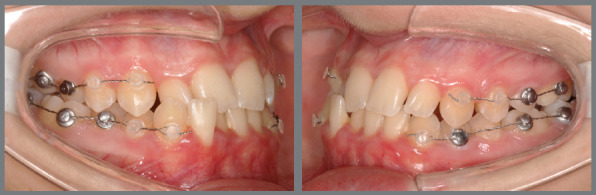
Image showing orthodontic buttons secured with ligature wire, providing additional stability and preventing the loss of accessories during surgery.

**Figure 5:**
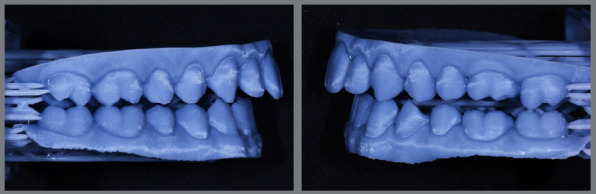
3D-printed occlusal models simulating the movements planned for orthognathic surgery. After scanning this new occlusal relationship, the clinician proceeds with the digital setup to design the aligners for post-operative treatment.

In orthodontic preparation for surgery using aligners, whether following the Conventional Orthognathic Protocol (COP) or the Surgery-First Approach (SFA), the primary goal is to efficiently and effectively perform dental decompensation and occlusal adjustments. In the COP, after dental decompensation is complete, the last three aligners should be passive and include button cutouts. At this stage, the surgeon refines the treatment plan based on updated orthodontic records, which helps determine the post-surgical occlusion and allows for the fabrication of surgical guides and post-surgical aligners. This step is crucial to ensure the correct execution of the surgical plan and the production of surgical splints and aligners for the post-surgical orthodontic phase. Before surgery, all aligner attachments should be removed to prevent interference with the surgical guides. Orthodontic buttons are then placed from molars to canines in both arches to enhance trans- and post-surgical stability (Fig 3). Additionally, the orthodontic buttons can be secured with ligature wires to reduce the risk of detachment and prevent the loss of orthodontic accessories during surgery (Fig 4). In the weeks leading up to surgery, it is recommended that the patient wear passive aligners, to stabilize the occlusion. The scan for fabricating the post-surgical aligners must account for the corrected post-surgical occlusion. After scanning the teeth, a bite is registered using a model simulating the surgical correction (Fig 5). This ensures that the aligners are ready for immediate use in the post-surgical orthodontic phase, facilitating a smooth transition from surgery to treatment, and preventing delays. Another option is to scan the teeth before surgery and register the bite afterward. In this case, the patient would wear passive aligners until the final aligners are fabricated. While this approach may result in the loss of a few weeks of accelerated movement, the benefit is that the aligners will be made based on the actual occlusion achieved during surgery. Both alternatives are viable, depending on the professionals’ preferences.

**Figure 6:**
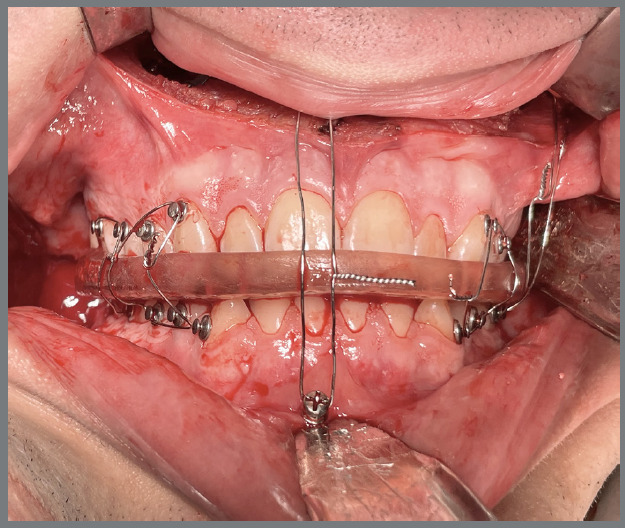
Surgical phase showing the trans-surgical guide in place, supported by skeletal anchorage and secured with metallic ligature wires.

In the SFA protocol, the first set of aligners should be passive, without attachments, and should include cutouts for buttons. The patient should start using these passive aligners about one week before surgery, to become familiar with their placement and removal. In both the SFA and COP, the first 10 post-surgical active aligners include cutouts for buttons on the molars and canines, to optimize stability during and after surgery. This design allows the aligners to be inserted and removed without interfering with surgical guides. Intermaxillary fixation screws are used during surgery to provide greater stabilization control. In some cases, temporary anchorage devices (TADs) may also be employed, positioned according to the patient’s needs and the clinical expertise of the professionals involved (Fig. 6).

## POST-OPERATIVE CARE

A primary concern in aligner-based surgical treatments is post-operative intermaxillary fixation. Unlike traditional fixed appliances, aligners lack the inherent stability provided by wires and brackets to ensure the maintenance of surgical outcomes. Studies have shown different approaches to overcome this limitation,^12^ ^,^ ^20^ ^-^ ^22^ including post-surgical guides, intermaxillary elastics, TADs, and buttons, to enhance occlusal stability and control dental movements. There are also variations in intermaxillary fixation protocols,^12^ with some approaches suggesting an initial use of guides or passive aligners for immediate stabilization, followed by a gradual transition to active aligners.

**Figure 7:**
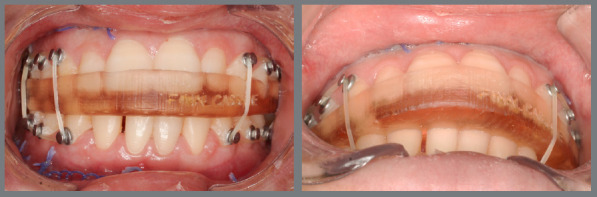
Intraoral photographs showing the use of the final surgical guide, employed for 2-3 weeks following orthognathic surgery.

Current guidelines vary regarding the minimum duration for wearing these guides,^12^ and the literature has not established the best approach for minimizing patient discomfort related to guides use and stabilization. Based on clinical experience, it is generally recommended that the guides and elastics be worn for at least 2 to 3 weeks post-surgery, to enhance stabilization and to support neuromuscular adaption (Fig 7). During this time, the guide is temporarily removed once a week for cleaning and occlusion assessment. This practice is particularly important in the SFA, due to the inherent stability challenges when using aligners. In the COA, depending on the orthodontist’s expertise and post-surgical stability, immediate orthodontic treatment can begin to optimize the regional acceleratory phenomenon (RAP). Patients are advised to follow a liquid or soft diet for the first two weeks, gradually transitioning to solid foods based on their comfort and healing progress. Most patients can resume regular eating within 6 to 8 weeks, once the maxillaries have sufficiently healed.

In surgical cases involving maxillary segmentation, the use of a palatal splint is recommended to support healing and maintain the expansion of the maxillary segments following surgery. This splint remains in place for at least three months, anchored securely to the teeth through the interdental papilla and contact points. In addition to stabilizing the repositioned segments, the splint helps control masticatory forces, which is essential for preventing the displacement or collapse of the segments. By managing these forces, the splint promotes a safer and more controlled recovery, ensuring the integrity of the maxillary expansion during the healing process.^20^

## ORTHODONTIC TREATMENT

**Figure 8:**
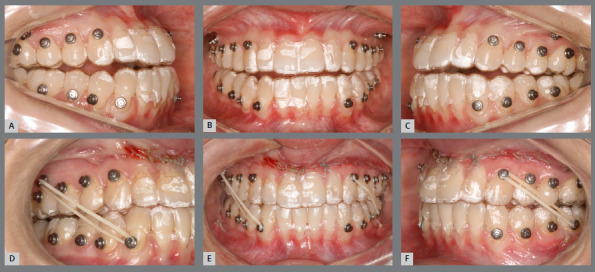
A,B,C) Images taken before the surgery. D,E,F) Images showing the condition one-month post-surgery.

As previously described, post-surgical dental movements can begin either immediately after surgery or following the use of occlusal guides for two to three weeks, depending on the level of occlusal stability achieved. Once active aligners are introduced, typically during the first three months of accelerated movement, they can be changed every five days if the aligners are well adapted and the patient experiences no discomfort. Intermaxillary elastics are used to stabilize the occlusion and assist in making necessary adjustments with the help of bonded buttons (Fig 8). After this initial phase, the standard protocol for aligners change is extended from 7 to 14 days, depending on the clinician’s preference.

The total orthodontic treatment time with aligners is comparable to that of fixed appliances, typically ranging from 12 to 24 months.^23^ ^-^ ^25^ To enhance post-operative control and patient comfort, it is recommended to refer patients to physiotherapy, with light exercises starting one week after surgery, and gradually increasing in intensity over the following weeks. Most patients resume regular activities within two weeks, with facial swelling significantly decreasing between the second and third weeks post-procedure. It is also common for aligner adjustments or additional aligners to be needed to fine-tune orthodontic finishing if the occlusion differs from the original plan.

## CLINICAL CASES: CONVENTIONAL ORTHOGNATHIC PROTOCOL (COP)

**Figure 9:**
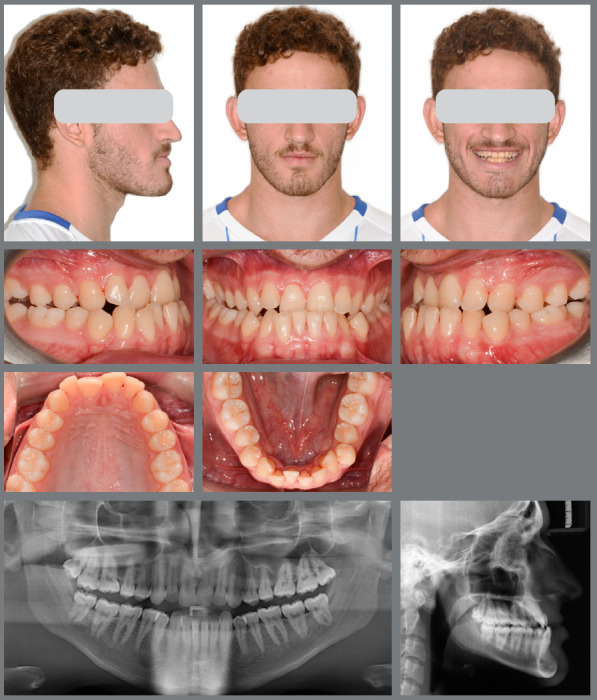
Pretreatment evaluation.

A male patient seeking orthodontic treatment, concerned about “misaligned teeth and an overly prominent chin.” His clinical examination revealed facial symmetry with midface depression, an increased chin-neck line, a thin upper lip, and an open nasolabial angle. The pogonion and lower lip were also projected forward in relation to the upper lip. Figure 9 illustrates the patient’s pre-treatment facial, dental, and radiographic characteristics. He exhibited a mesocephalic craniofacial pattern (SN.GoGn = 21.9°, FMA = 14.9°, Y-axis = 56.0°), along with a significant skeletal Class III discrepancy (ANB = -7.6°; Wits = -13.2°). Molars and canines were in a Class III relationship, with anterior crossbite and retroinclined lower incisors.

**Figure 10:**
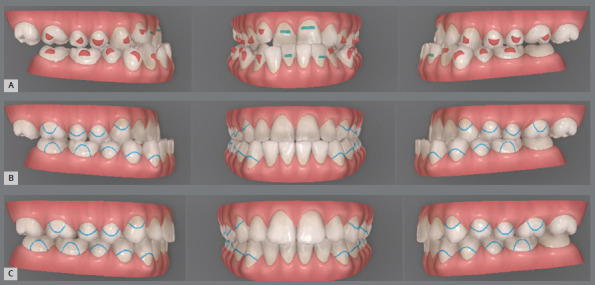
Images of the digital setup for the pre-surgical orthodontic phase. A) Occlusion before treatment. B) Occlusion before simulating surgical advancement. C) Occlusion after simulating the surgical advancement.

**Figure 11:**
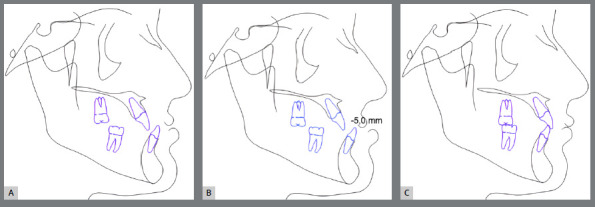
2D Treatment planning. A) Occlusion before surgery. B) Occlusion after simulating dental decompensations. C) Occlusion after simulating orthodontic treatment completion.

The initial digital setup focused on aligning and leveling the maxillary arch, maintaining the current position of the incisors, while incorporating a minor 2-mm transverse expansion. In the mandibular arch, alignment and leveling were performed to address crowding and flatten the curve of Spee, achieved through a 2-mm projection of the incisors and expansion, without the need for interproximal reduction. Maxillary advancement to correct the negative overjet was simulated only in the final aligner, as it was intended to mirror the planned orthognathic surgery (Fig 10). Prior to the first VSP session with the surgeon for movement confirmation, attachments were removed and buttons were placed from the molars to the canines on the last three aligners (Fig. 11).

**Figure 12:**
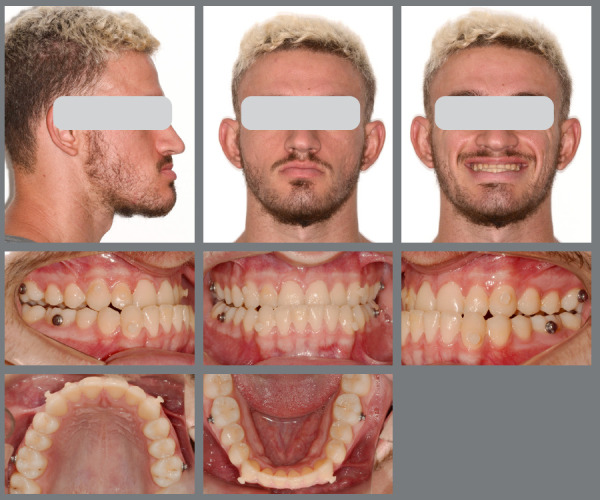
Facial and intraoral images at the end of orthodontic preparation.

**Figure 13:**
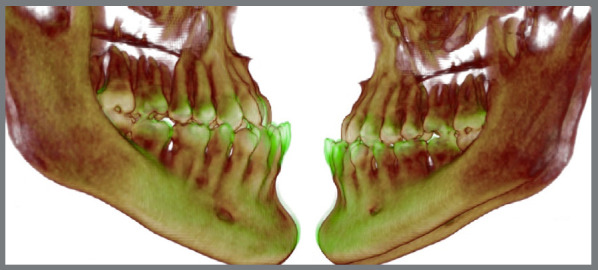
Superimposed images of pre-orthodontic treatment CBCT scans with orthodontic movements in the pre-surgical phase (in green), highlighting the dental changes achieved.

**Figure 14:**
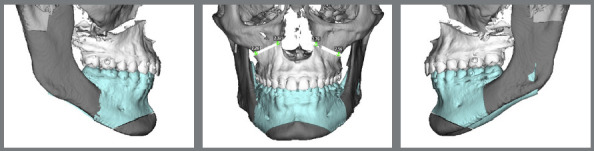
Virtual Surgical Planning (VSP) simulation demonstrating post-surgical maxillary advancement (+7 mm) and mandibular setback (-5 mm).

After the treatment plan was accepted, 27 pairs of aligners were prescribed. The patient was instructed to wear the aligners for 20-22 hours per day, removing them only for eating and oral hygiene. Aligners were changed every 10 days, with clinical appointments scheduled every 4 weeks. Extrusion attachments were added to the posterior teeth to better control the overbite, while power ridges were used to improve the projection of the lower incisors. After 9 months of dental decompensation (Fig 12), a new VSP was performed to confirm the surgical movements, using a new scan for post-surgical treatment planning (Figs 13 and 14).

For post-surgical treatment, sequential mesialization of the mandibular dentition was planned to achieve a Class I relationship in molars and canines, along with extrusion of the maxillary molars and premolars. In addition, the projection of the incisors was planned with the addition of overcorrection of the anterior vestibular torque, as well as appropriate coordination of the arches. Buttons were placed on the first molars and canines. Four refinement stages were necessary, leading to a 24-month post-surgical treatment period, due to difficulties in controlling posterior extrusion and correcting the posterior open bite, a common challenge in aligner treatments.

**Figure 15:**
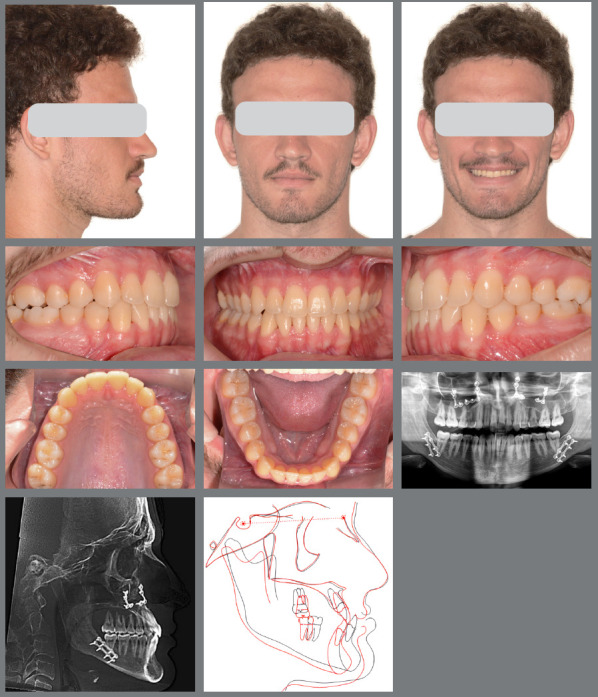
Facial and intraoral images, and radiographs at the final post-treatment stage.

**Figure 16:**
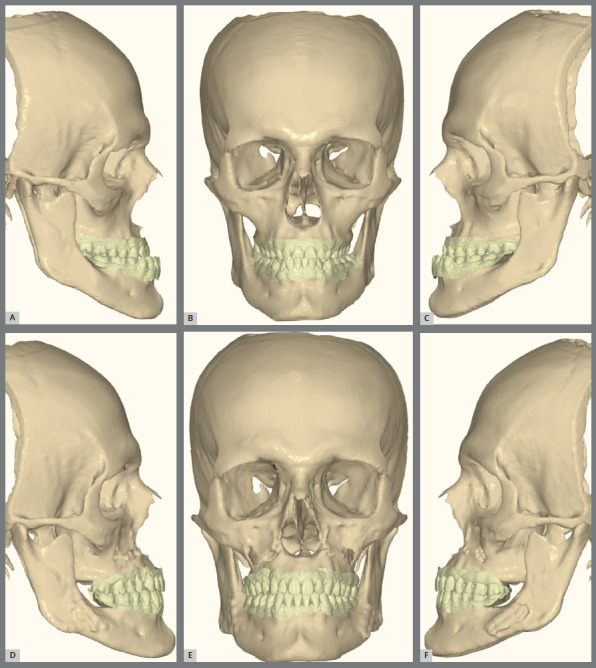
CBCT images: **A-C)** pre-operative malocclusion, **D-**F) outcome after orthodontic and surgical treatment.

**Figure 17:**
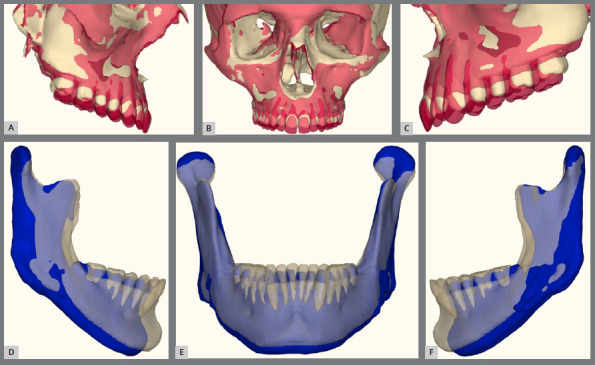
A-C) Maxillary superimpositions showing pre-surgical (beige) and post-treatment (pink) positions. D-F) Mandibular setback illustrated with pre-surgical (beige) and post-treatment (blue) superimpositions.

The facial results demonstrated excellent harmony between the lips and pogonion, with a well-balanced nasolabial angle and an overall harmonious profile. From a dental perspective, the treatment was also successful, achieving Class I occlusion in both canines and molars, with proper overbite and overjet, effective transverse control, and satisfactory root parallelism (Fig 15). The inclinations of both maxillary and mandibular incisors were also properly adjusted. The 3D superimposition images confirmed the treatment results (Figs 16 and 17).

## SURGERY-FIRST APPROACH (SFA)

**Figure 18:**
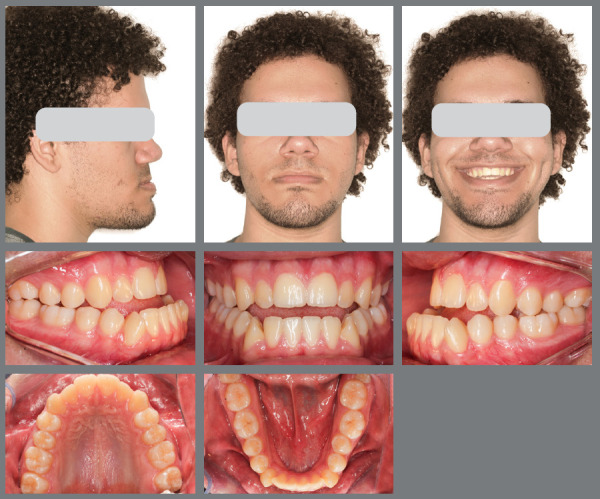
Pre-treatment facial and intraoral images.

A male patient seeking orthodontic treatment due to concerns about his excessively prominent chin. He presented with slight facial asymmetry to the right, an increased lower third of the face, a pronounced midface depression, and an accentuated chin-neck line. The upper lip was thin, while the lower lip was thick, with a closed nasolabial angle and an obtuse mentolabial angle. Cephalometric analysis revealed a skeletal Class III relationship (ANB = -6°; Wits = -16 mm) and a mesocephalic pattern (SN.GoGn = 29°; FMA = 30°; Y-axis = 56°). Molar and canine relationships were Class III, with -7 mm overjet, anterior open bite, crossbite, and mild dental compensations (Fig 18).

The SFA was chosen primarily due to the patient’s aesthetic concerns and social interaction issues. Additionally, the predicted post-operative occlusion was stable, allowing the surgery to proceed without prior orthodontic preparation. Occlusal features that made this patient an ideal candidate for SFA included a mild curve of Spee, well-positioned maxillary incisors, slightly retroclined mandibular incisors, no significant occlusal plane inclination, mild crowding of the lower anterior teeth, minimal dental midline asymmetry, and moderate transverse discrepancy.

**Figure 19:**
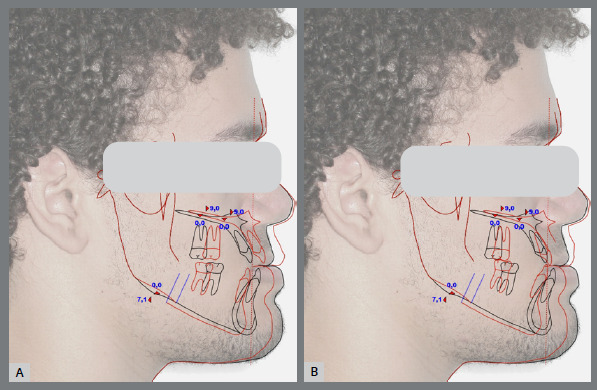
2D treatment planning: **A)** pre-orthodontic phase, outlining the surgical treatment objective; **B)**post-orthodontic phase, showing the final treatment result.

**Figure 20:**
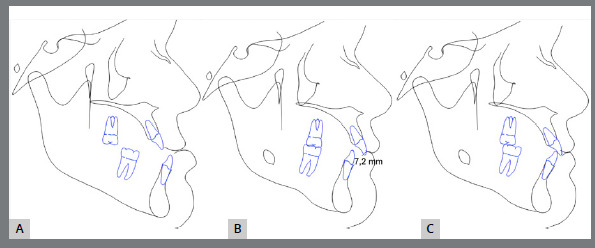
2D treatment planning: A) occlusion prior to surgery; B) occlusion after simulating the surgical advancement; C) occlusion following the simulated orthodontic treatment.

**Figure 21:**
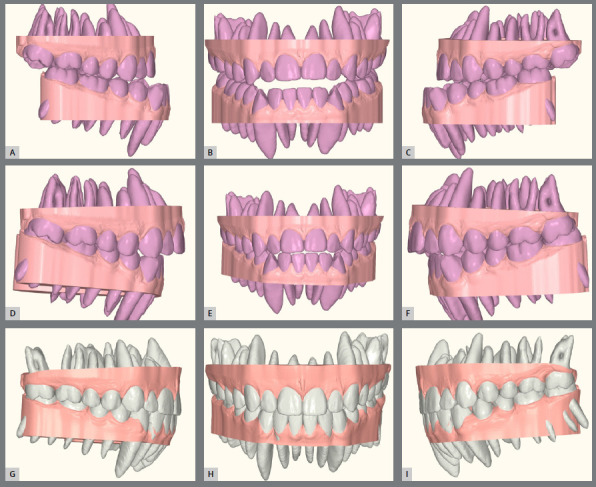
Digital setup images: **A-C**) pre-surgical occlusion; **D-**F) simulated occlusion after surgical advancement; **G-I**) post-treatment occlusion.

The treatment plan began with VSP, integrating pre-operative CBCT and intraoral scans of the maxillary and mandibular arches. In the 2D planning, maxillary advancement of (+9 mm) and mandibular setback of (-7 mm) were simulated, along with the necessary decompensation in anterior tooth inclinations, which included a mild projection of the maxillary incisors (4.0°) and a significant projection of the mandibular incisors (11.0°) (Figs. 19 and 20). The virtual setup planned an 11-mm advancement with aligner 2, simulating the surgical “jump”, without requiring interproximal reduction (IPR). Additionally, posterior distalization and a 2-mm retraction of the maxillary incisors were incorporated to address the predicted post-surgical Class II relationship (Fig. 21).

**Figure 22:**
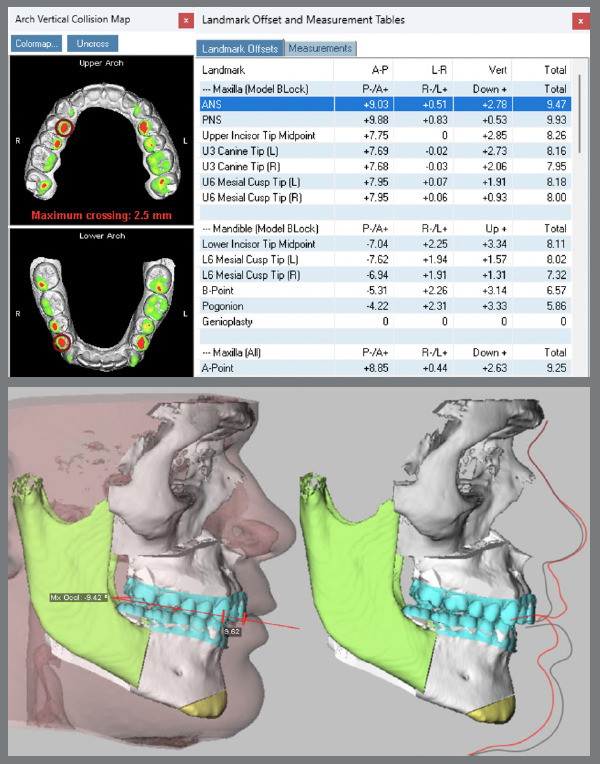
3D Surgical planning.

The upper arch was leveled and aligned according to the lower arch. Aligners 1 to 10 were designed with cutouts for button placement on the molars, premolars, and canines. From aligner 11 onward, specific cutouts were added to optimize control over tooth movements. The main challenge in this case was correcting the rotations of the lower canines, due to limited control over complex rotations with aligners. The strategy involved overcorrecting rotations and projecting the mandibular teeth. In the 3D planning, surgical movements refinements were made, the pre-established directions were maintained, ensuring the coherence of the initial plan with the necessary corrections (Fig 22). Once the refinements were finalized and approved, the aligners were ordered.

**Figure 23:**
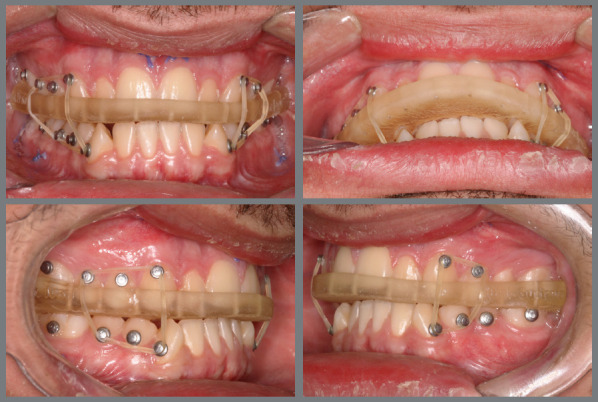
Intraoral photographs showing the use of the final surgical guide, utilized for three weeks post-surgery. Observe the use of elastics for better fitting and adaptation of the teeth to the guide.

**Figure 24:**
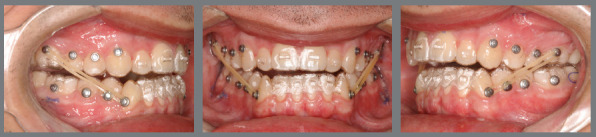
Intraoral photographs demonstrating the discontinuation of the guide’s use and the beginning of orthodontic treatment. Class III intermaxillary elastics were employed to stabilize surgical movements and control postoperative relapse.

After the surgery, the patient wore a surgical splint for three weeks, to ensure initial occlusal stabilization in cases of SFA treated with aligners (Fig 23). Orthodontic treatment followed, involving the use of intermaxillary elastics, alongside changing aligners every five days during the first three months (Fig. 24). This accelerated schedule leveraged the RAP effect. Initially, the treatment plan included 23 maxillary aligners and 28 mandibular aligners. After six months, a new scan was conducted for refinement. The objectives included a 5º projection of the maxillary incisors, mandibular incisor intrusion, and posterior extrusion, supported by bite ramps on the palatal surfaces of the maxillary anterior teeth. To prevent relapse and overcorrect the Class III malocclusion, distalization of the mandibular posterior teeth on both sides was proposed. These objectives were successfully achieved through two refinement stages.

**Figure 25:**
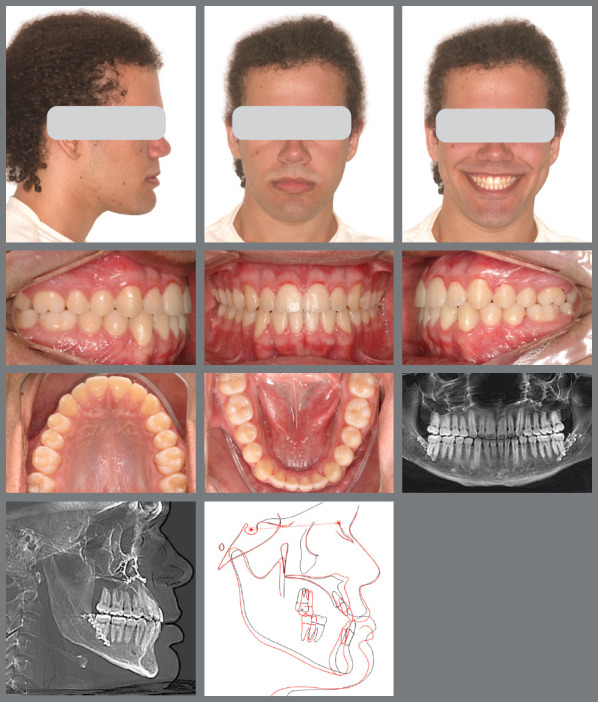
Facial and intraoral images and radiographs at the final post-treatment stage.

**Figure 26:**
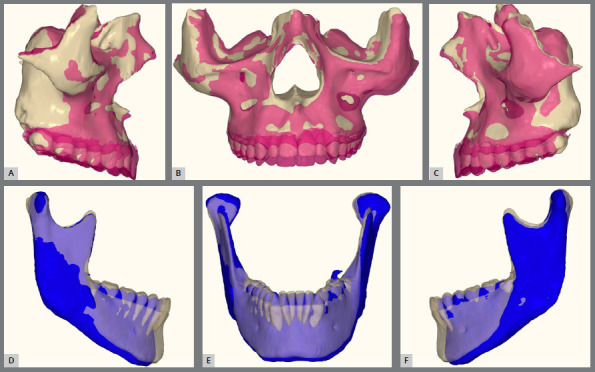
A-C) Maxillary superimpositions showing pre-surgical (beige) and post-treatment (pink) positions. D-F) Mandibular superimpositions with pre-surgical (beige) and post-treatment (blue) positions.

The results achieved were satisfactory from both facial and dental perspectives, leading to high patient satisfaction. The orthodontic treatment lasted 22 months post-surgery. By the end of treatment, the patient’s facial aesthetics improved notably, particularly through the reduction of midface depression and the enhancement of upper lip thickness due to maxillary advancement. Importantly, these improvements were accomplished without altering the nasolabial angle or nasal tip projection (Fig. 25). The patient now demonstrates better tooth exposure when smiling, in contrast to the prior open bite and inverted smile. Additionally, Class I occlusion in canines and molars, along with proper overjet and alignment, was achieved. The 3D superimpositions confirmed the success of the treatment (Fig 26).

## CONCLUSION

This study described protocols for orthodontic-surgical treatments using clear aligners. The presented cases demonstrated that achieving final results consistent with the initial treatment plan is feasible. While clear aligners provide appealing features, the practitioner becomes more dependent on patient compliance. Furthermore, certain movements remain challenging and less predictable with aligner treatment. Although the technique is promising, well-conducted clinical studies are necessary to enhance the quality of available evidence, assessing whether facial and occlusal outcomes are comparable to those achieved with fixed appliances.
